# Proportions of Basement Membrane Proteins in Cerebrovascular Smooth Muscle Cells After Exposure to Hypercapnia and Amyloid Beta

**DOI:** 10.3390/cells14080614

**Published:** 2025-04-18

**Authors:** Jennifer M. Dewing, Abby Keable, Alexandru Laslo, Laura Chinezu, Adrian Ivanescu, J. Arjuna Ratnayaka, Raj Kalaria, Mark Slevin, Ajay Verma, Roxana O. Carare

**Affiliations:** 1 Faculty of Medicine, University of Southampton, Southampton SO17 1BJ, UK; abbykeable@gmail.com (A.K.); j.ratnayaka@soton.ac.uk (J.A.R.); r.o.carare@soton.ac.uk (R.O.C.); 2 British-Romanian Academic Institute of Neuroscience (BRAIN), University of Medicine, Pharmacy, Science and Technology “G.E.Palade” Targu Mures, 540142 Targu-Mures, Romania; alexandru.laslo@gmail.com (A.L.); laura_chinezu@yahoo.com (L.C.); dr_adrian_ivanescu@yahoo.com (A.I.); mark.slevin@umfst.ro (M.S.); 3 Neurovascular Research Group, Translational and Clinical Research Institute, Newcastle University, Campus for Ageing & Vitality, Newcastle upon Tyne NE4 5PL, UK; raj.kalaria@newcastle.ac.uk; 4 Formation Venture Engineering Foundry, Boston, MA 02494, USA; ajay@formationve.com

**Keywords:** amyloid beta, basement membranes, cerebral amyloid angiopathy, hypercapnia, intramural periarterial drainage

## Abstract

Vascular basement membranes (BMs), composed of laminins, collagen IV, fibronectin, and perlecan, are secreted by endothelial cells, pericytes, smooth muscle cells (SMCs), and astrocytes. In the brain, amyloid beta (Aβ) is eliminated along cerebrovascular BMs of capillaries and arteries as intramural periarterial drainage (IPAD). Ageing modifies vascular BMs, impairing IPAD and leading to Aβ deposition as cerebral amyloid angiopathy. To better understand the molecular determinants of IPAD in ageing, we quantified the relative abundance of BMs secreted by human-derived cerebral endothelial cells, pericytes, brain vascular SMCs, and astrocytes in vitro. We then assessed BM protein levels in SMCs under hypercapnia (8% CO_2_) as a model of vascular ageing, with and without Aβ exposure. Of the four cell types, we found SMCs secreted the highest levels of fibronectin, laminin, and perlecan, whilst pericytes secreted the highest levels of collagen IV. Hypercapnia increased the expression of collagen IV and fibronectin in SMCs but decreased the expression of laminin. The expression of perlecan increased under hypercapnia, but only in the presence of Aβ. This work highlights the varying compositions of vascular BMs and the dynamic differential responses of SMCs to Aβ and hypercapnia, helping to elucidate the age-related changes that impair IPAD in cerebral vessels.

## 1. Introduction

**Figure 1:**
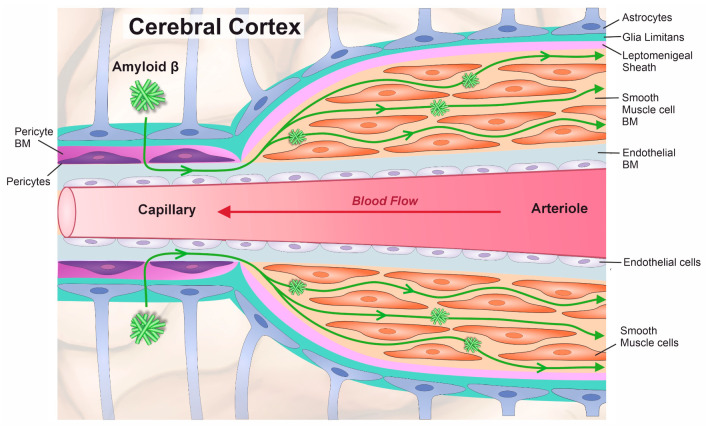
Route of Aβ drainage along basement membranes (BM) of cerebral capillaries and arteries as intramural periarterial drainage (IPAD). IPAD occurs in the opposite direction to blood flow and the motive force is provided by the spontaneous contraction of smooth muscle cells. Apart from the basement membrane of the endothelium (light blue), there are basement membranes surrounding smooth muscle cells (orange) and basement membranes of the astrocytes also known as glia limitans (green). Pericytes are embedded in basement membranes (purple). A sheath of leptomeninges represents the adventitia of arteries but is not present at the capillary level. With ageing, the structure of the basement membranes changes, impairing IPAD and increasing Aβ deposition as a cerebral amyloid angiopathy (CAA).

Most cells are surrounded by a supporting scaffold of extracellular glycoproteins and proteoglycans that offer anchorage and structural support, forming dense sheets known as basement membranes (BMs). In the absence of traditional lymphatic vessels, the BMs of cerebral capillaries and arteries represent a conduit for the drainage of interstitial fluid and soluble amyloid beta (Aβ) as intramural periarterial drainage (IPAD) (Figure 1) [1,2,3]. The motive force for IPAD is provided by the spontaneous contraction of vascular smooth muscle cells (SMCs) [4].

Ageing leads to arteriolosclerosis, whereby changes in the composition of BMs, a decline in the bioavailability of nitric oxide, and a reduction in the strength of contractions of vascular SMCs lead to hypoperfusion, hypercapnia, and an impairment of IPAD [5,6,7]. The age-associated failure of IPAD leads to the accumulation of Aβ within BMs of cerebral capillaries and arteries as cerebral amyloid angiopathy (CAA), identifiable in over 80% of Alzheimer’s disease (AD) cases [8,9]. In human CAA, the accumulation of Aβ begins between the layers of cerebral vascular SMCs in arterioles and the leptomeningeal arteries, eventually leading to the destruction of the SMCs [10]. This proposed sequence, based on observations in human post-mortem brains and brains of transgenic mice overexpressing Aβ, suggests that soluble Aβ drains along the BMs of vascular SMCs [11]. The BMs secreted by vascular SMCs are morphologically distinct from the endothelial BM [12]. The exact composition of BMs varies in a temporal and tissue-specific manner [13], but it is highly conserved and consists of a core set of proteins. Typically, each BM is composed of laminin, collagen IV, nidogen, and a heparan sulphate proteoglycan such as perlecan or agrin [14]. Fibronectin is also an abundant, ubiquitous extracellular matrix (ECM) component that acts as a critical mediator of cell–ECM interactions by bridging structural components of the BM with cell surface receptors, such as integrins [15,16]. Following deposition by resident cells of the cerebrovasculature, laminin and collagen IV self-assemble into three-dimensional networks that interconnect with nidogen and perlecan [17]. Collagen IV is essential for BM stability, resilience, and vascular integrity, with mouse models exhibiting aberrantly spliced collagen IV, developing pathology reminiscent of small vessel disease [9,18]. Laminin is the most abundant non-collagenous BM protein and it exists as distinct isoforms depending on the vessel type [9,19]. Laminin α2 isoform-knockout mice exhibit a defective blood–brain barrier (BBB), suggesting an essential role of laminin in BBB integrity [20]. Perlecan is one of the most abundant heparin sulphate proteoglycans (HSPGs), and once embedded in the collagen IV/laminin network, perlecan helps to maintain BM integrity as well as bind important growth factors [21]. The production and secretion of these aforementioned BM proteins is provided by the cells of the cerebrovasculature, although the proportional contribution of each cell type remains unknown. Nonetheless, it is widely accepted that the precise levels, distribution, and interaction of BM proteins mutually support the health of and communication between the cells of the cerebrovasculature.

Ageing leads to reduced respiratory response and cerebrovascular reactivity to hypercapnia; thus, cells of ageing cerebrovasculature are likely exposed to higher levels of CO_2_, [22,23] and it remains unknown how the BMs of cerebrovascular cells may respond to these conditions. A better understanding of the BM composition of cerebral vascular cells, in particular SMCs and their response to Aβ and hypercapnia as a model of hypoperfusion and ageing, will offer insights into the vascular extracellular changes that influence IPAD and contribute to CAA. Here, we tested the following hypotheses: (a) the composition of BMs of human endothelial cells, pericytes, astrocytes, and SMCs differ from each other when grown in culture under normocapnic conditions (5% CO_2_) and (b) exposure to Aβ 1–40 or Aβ 1–42 and hypercapnia (8% CO_2_) modifies the composition of BMs of SMCs [24].

## 2. Materials and Methods

### 2.1. Amyloid Beta Preparation

The HiLyte 555 Aβ 1–40 is a synthetic protein provided by AnaSpec (Fremont, CA, USA) (#AS-60492-01), with a fluorescent tag that allows for observation by fluorescent microscopy. The sequence for this protein was as follows: HiLyte Fluor 555-DAEFRHDSGYEVHHQKLVFFAEDVGSNKGAIIGLMVGGVV. This corresponds to the human Aβ which is 40 amino acids in length and commonly found in CAA [25]. The HiLyte 647 Aβ 1–42 was also provided by AnaSpec (#AS-64161) and has the following sequence: HiLyte Fluor 647-DAEFRHDSGYEVHHQKLVFFAEDVGSNKGAIIGLMVGGVVIA. This sequence corresponds to the human Aβ commonly found in senile plaques and is 42 amino acids in length [26]. Both products were supplied as a lyophilized powder and reconstituted in 1% ammonium hydroxide to a final concentration of 200 mM before being aliquoted and stored at −80 °C. Aliquots were only thawed immediately prior to use and were added directly to pre-warmed media to obtain a final concentration of 100 nM.

### 2.2. Cell Cultures

Human astrocytes (HAs), human brain vascular smooth muscle cells (HBVSMCs), and human pericytes (HP) were purchased from Sciencell (#1800, #1100 and #1200, respectively). The human cerebral microvascular endothelial cell line (hCMEC/D3) was purchased from Sigma Millipore (#SCC006). Cells were grown according to the manufacturer’s instructions and maintained in specialised media. Cells at passage number < 10 were used for experiments to guarantee the retention of cell properties, as stated by the manufacturers. The HAs and HBVSMCs were guaranteed to retain properties for 10 population doublings and the HPs for up to 15, hence the cells were used within these parameters for all experiments. Media for HAs, HBVSMCs and HPs were obtained from Sciencell (Carlsbad, CA, USA) (#1801, #1101 and #1201), whilst hCMEC/D3 was maintained in EndoGRO basal medium and a supplement kit (SCME-004, Merck Millipore, Darmstadt, Germany) enhanced with 1ng/mL basic fibroblast growth factor (F0291, Sigma) and 1% penicillin–streptomycin (15140-122, Thermo Fisher Scientific, Cambridge, UK). Cultures were maintained in a humidified environment (5% CO_2_/95% air) at 37 °C. For the analysis of basement membrane protein deposition by each cell type, HAs, HPs, and HBVSMCs were plated from 24-well plates onto 1 mm round glass coverslips coated with poly-L-lysine or collagen I for hCMEC/D3 (*n =* 4). The initial cell seeding density was 0.5 × 10^5^ cells per well in 1 mL of media, and cells were left to grow for 72 h before fixation and immunostaining. For hypercapnia/Aβ experiments, HBVSMC were plated in 12 well plates onto 1 mm round glass coverslips coated with poly-L-lysine at a density of 0.1 × 10^6^ cells in 1 mL smooth muscle medium (#1101 Sciencell) and left for 4 h to attach. The cell media was then replaced with either fresh media or media supplemented with 100 nM Aβ1–40 or Aβ 1–42. Cells were incubated for 72 h in separate incubators set at either 5% CO_2_/95% air (normocapnic) or 8% CO_2_/92% air (hypercapnic) conditions (*n* = 3). Our pilot data showed 100 nM Aβ to be the lowest concentration that elicited a cellular response.

### 2.3. Immunocytochemistry for BM Components

Further, 72 h after seeding, cells were fixed in 1 mL 4% PFA for 10 min at room temperature and immunostained for BM proteins: collagen IV, laminin, fibronectin, and perlecan, as described previously [24]. Briefly, cells were rinsed three times with phosphate-buffered saline (PBS) before quenching residual PFA with 100 nM glycine for 10 min. Cells were washed three times in PBS and incubated overnight at 4 °C in 3% bovine serum albumin (BSA) for 1 h at room temperature. The BSA was removed and cells were incubated with antibodies against collagen IV (ab6586, Abcam, Cambridge, UK 1:400), laminin (L9393, Sigma, Burlington, MA, USA 1:200), fibronectin (F3648, Sigma, Burlington, MA, USA, 1:400), or perlecan (sc-25848, Santa Cruz, Dallas, TX, USA, 1:400). Cells were washed three times with PBS and incubated with goat anti-rabbit Alexa Fluor 555 conjugated secondary antibody for 1 h at room temperature. For hypercapnia/Aβ experiments, HBVSMCs were incubated with goat anti-rabbit Alexa Fluor 488 conjugated secondary antibody, due to the presence of HiLyte 555 Aβ 1–40 in a subset of the cells. Cells were then washed three times before 2 µg/mL DAPI (D1306, Thermo Fisher, Cambridge, UK) was applied for 10 min to stain cell nuclei. Cells were rinsed three times with PBS and the coverslips inverted and mounted onto slides with Mowiol (81381, Sigma, Burlington, MA, USA) containing Citifluor (AGR1320, Agar Scientific, Rotherham, UK). An isotype control was used to estimate the non-specific binding of target primary antibodies as a result of Fc binding. Cells were treated with an IgG isotype control (ab37415, Abcam, Cambridge, UK) at the same protein concentration as the primary antibody. In addition, a negative control was also carried out whereby the primary antibody was omitted to assess any non-specific reactivity of the secondary antibody.

### 2.4. Imaging and Analysis

Cells were imaged with a Leica SP8 confocal microscope. Isotype and negative control cells were used to set the gain and exposure to adjust for background and non-specific staining. Four non-overlapping z-stacks (16 µm stack, 1 µm per slice) were captured per coverslip (each an area of 0.1 mm^2^), with a minimum of three coverslips per experiment group. Maximum projection Z-stack TIF images were analysed using ImageJ software, (version 1.54g) (NIH, Bethesda, MD, USA) to quantify the number of nuclei from DAPI staining and the arbitrary fluorescence area of antibody staining using image thresholding (24, 255) of the desired colour channel. The fluorescence area was normalised to the number of cells by dividing the area of fluorescence by the number of nuclei per image.

### 2.5. MTS Assay

The CellTiter 96 AQueous one solution cell proliferation colorimetric assay kit (G3582, Promega, Hampshire, UK) was used to assess HBVSMC viability in the presence of Aβ and under hypercapnic conditions. Three timepoints were chosen to monitor cell proliferation over a time course: 2 h (day 0), 24 h (day 1), and 72 h (day 3). Cells were seeded onto poly-L-lysine coated wells at a density of 5 × 10^3^ cells per well on a black 96-well plate and cultured in smooth muscle medium containing either no Aβ, 100 nM Aβ 1–40, or 100 nM Aβ 1–42 at 37 °C under normocapnia (5% CO_2_, 95% air) or hypercapnia (8% CO_2_, 92% air) conditions. At each timepoint, 20 μL of MTS reagent was added to each well and the plate was incubated in the dark for 90 min. Absorbance was measured at 490 nm with a SPECTROstar Nano plate reader (BMG Labtech, Aylesbury, UK). Wells containing only media without cells were used to determine the baseline absorbance, the average value of which was subtracted from the test samples. All samples were run in triplicate and repeated three times (*n* = 3).

### 2.6. Data Analysis and Statistics

For the assessment of the BM composition in all four cell types, the four mean fluorescent values of each BM protein, calculated using ImageJ from four separate confocal images of each sample and normalised to cell number, were averaged (*n* = 4). Differences in the mean fluorescence of each BM protein were compared between cell types and analysed using a two-way ANOVA and Tukey Post Hoc multiple comparisons statistical analysis using Graph Pad Prism 10 software. For the assessment of the BM composition in HBVSMCs in response to hypercapnia and Aβ exposure, the four mean fluorescent values for each BM protein, per sample, calculated using ImageJ and normalised to cell number, were averaged (*n* = 3). Differences in mean fluorescence of each BM protein were compared between treatment conditions and analysed using a two-way ANOVA and Tukey Post Hoc multiple comparisons statistical analysis using Graph Pad Prism 10 software. For the MTS assay, the absorbance values of triplicate readings were averaged per sample (*n* = 3). Differences in mean absorbance were compared between treatment groups and analysed using a two-way ANOVA and Tukey Post Hoc multiple comparisons statistical analysis using Graph Pad Prism 10 software. For all analyses, *p* values above 0.05 were considered significant.

## 3. Results

### 3.1. Smooth Muscle Cells Produce the Highest Total Amount of Basement Membrane Proteins

**Figure 2:**
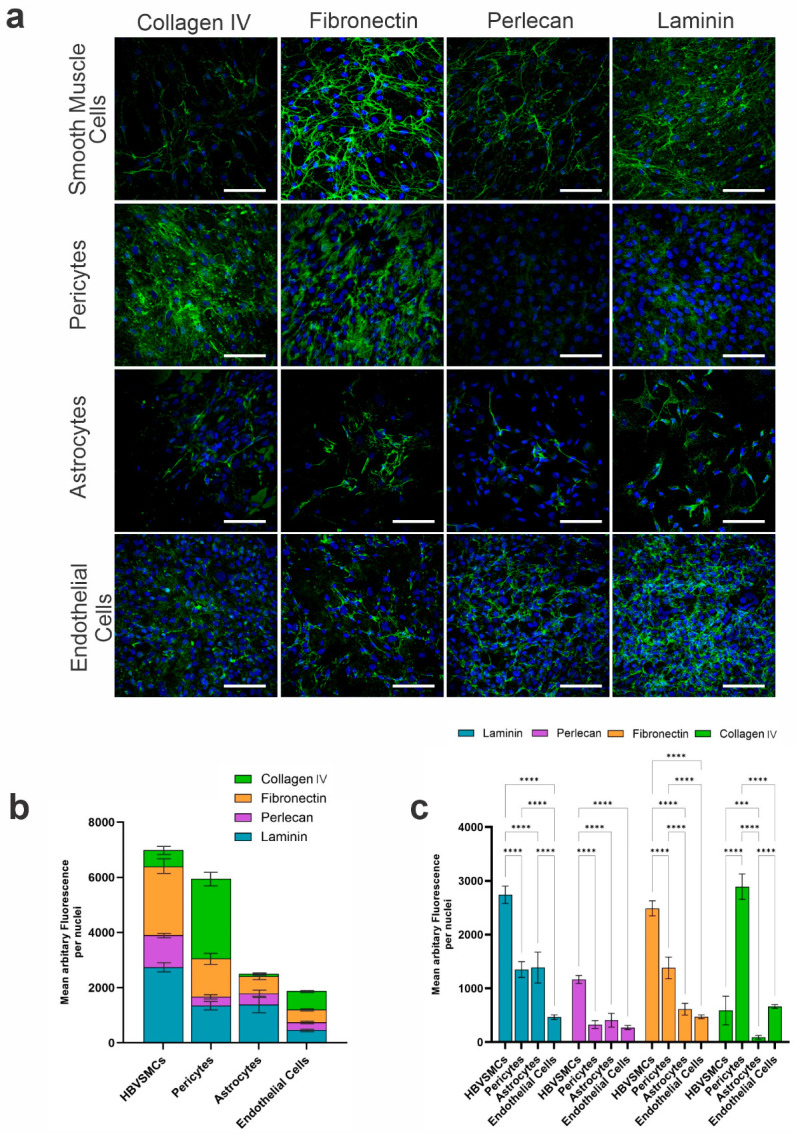
Relative abundance of basement membrane proteins in cerebrovascular cells measured by immunocytochemistry. (**a**) Representative confocal microscopy images of human astrocytes, endothelial cells (hCMEC/D3), pericytes, and human brain vascular smooth muscle cells (HBVSMCs) grown in culture for 72 h and immunostained for basement membrane proteins collagen IV, laminin, fibronectin, and perlecan, shown in green, and counterstained with the nuclear stain DAPI, shown in blue. Scale bar = 100 µm. (**b**) The mean fluorescence area per nuclei of each basement membrane protein was quantified and plotted as a stacked bar graph for each cell type (*n* = 4 per cell type, per protein). (**c**) Statistical significance between the mean fluorescence area per nuclei of each basement membrane protein between cell types (*n* = 4). *** *p* = 0.0001, **** *p <* 0.0001.

Qualitative comparison of the BM protein immunofluorescence levels from the various cerebrovascular cells showed that HBVSMCs produced the greatest amount of fibronectin, perlecan, and laminin compared to the other cell types (Figure 2a). In all cell types, a fibrous expression pattern of varying degrees was observed for fibronectin and perlecan, whilst laminin exhibited a more punctate pattern for astrocytes and pericytes. Similarly, the expression pattern of collagen IV was distinct in endothelial cells, with a more speckled appearance than the other cell types. Quantification of BM protein expression levels across cell types revealed that HBVSMCs expressed 73% more BM proteins (6986 mean total arbitrary fluorescence (AF)) relative to ECs (1876 mean total AF), 64% more than HAs (2503 mean total AF), and 14% more than pericytes (5954 mean total AF) (Figure 2b,c).

### 3.2. Perlecan Levels Were Consistent Between Pericytes, Astrocytes, and Endothelial Cells

HBVSMCs expressed significantly greater levels of perlecan (1165 mean AF) compared to HPs (327 mean AF), HAs (408 mean AF), and ECs (272 mean AF) (*p <* 0.001). No significant difference was observed between HPs, HAs, and ECs, suggesting these cell types express similar amounts of perlecan (*p* ≥ 0.05) (Figure 2c).

### 3.3. Pericytes and Astrocytes Express Similar Amounts of Laminin

HBVSMCs expressed significantly greater levels of laminin (2741 mean AF) compared to HPs (1351 mean AF), HAs (1389 mean AF), and ECs (466 mean AF) (*p <* 0.001). No significant difference was observed between HPs and HAs, suggesting that they express similar amounts of laminin (*p* = 0.9844) (Figure 2c).

### 3.4. Astrocytes and Endothelial Cells Express Similar Amounts of Fibronectin

HBVSMCs expressed significantly greater levels of fibronectin (2488 mean AF) compared to HPs (1382 mean AF), HAs (614 mean AF), and ECs (473 mean AF) (*p <* 0.001). No significant difference was observed between HAs and ECs, suggesting that they express similar amounts of fibronectin (*p* = 0.5488) (Figure 2c).

### 3.5. Pericytes Expressed the Highest Amount of Collagen IV

Whilst HBVSMCs expressed the highest amount of fibronectin, perlecan, and laminin, collagen IV was expressed at the greatest levels in pericytes, with more than four times more collagen IV in HPs (2892 mean AF) than HBVSMCs (590 mean AF) and ECs (664 mean AF) (Figure 2b,c). Astrocytes expressed very low levels of collagen IV (91 mean AF) compared to the other cell types. Statistical analyses (two-way ANOVA) confirmed that collagen IV levels were significantly different between all cell types (*p <* 0.0001), except for HBVSMCs and ECs (*p* = 0.8969), suggesting that these two cell types produce similar amounts of collagen IV (Figure 2c).

### 3.6. Hypercapnia and Aβ Exposure Do Not Affect HBVSMC Viability

**Figure 3:**
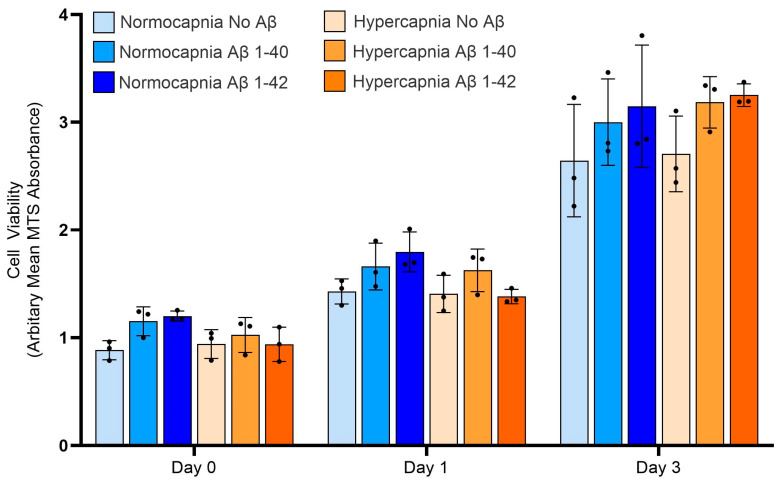
Cell viability of HBVSMCs cultured under normoxia verses hypercapnia, and in the presence/absence of amyloid beta. An MTS assay was used to assess the viability and metabolic activity of HBVSMC cultures exposed to normoxia or hypercapnia conditions, in the presence/absence of 100 nM Aβ40 or Aβ42 for 72 h. The mean MTS absorbance was calculated from triplicate wells for each condition over three experimental runs (*n* = 3). Black dots represent mean MTS from each run.

Cerebrovascular SMCs are the source of the motive force required for intramural periarterial drainage, and the BMs of these cells represent the conduits for IPAD [4]. Therefore, we wanted to investigate how hypercapnia (8% CO_2_) and exposure to Aβ influence the levels of BM proteins in these cells. Firstly, using an MTS colorimetric assay, we determined the viability of HBVSMCs over 72 h following exposure to hypercapnia and Aβ to determine whether any potential changes observed in BM composition was related to the health of the cells (Figure 3). Cell viability and metabolism, as measured by arbitrary absorbance, increased in all conditions over the 3 days, confirming cell proliferation. Overall, there was no significant difference in the viability of cells cultured at 5% versus 8% CO_2_, or in the presence/absence of Aβ 1–40 and Aβ 1–42. These findings suggests that physiological concentrations of Aβ do not adversely affect the metabolism and viability of HBVSMCs.

### 3.7. Hypercapnia Increased Collagen IV Expression in HBVSMCs

**Figure 4:**
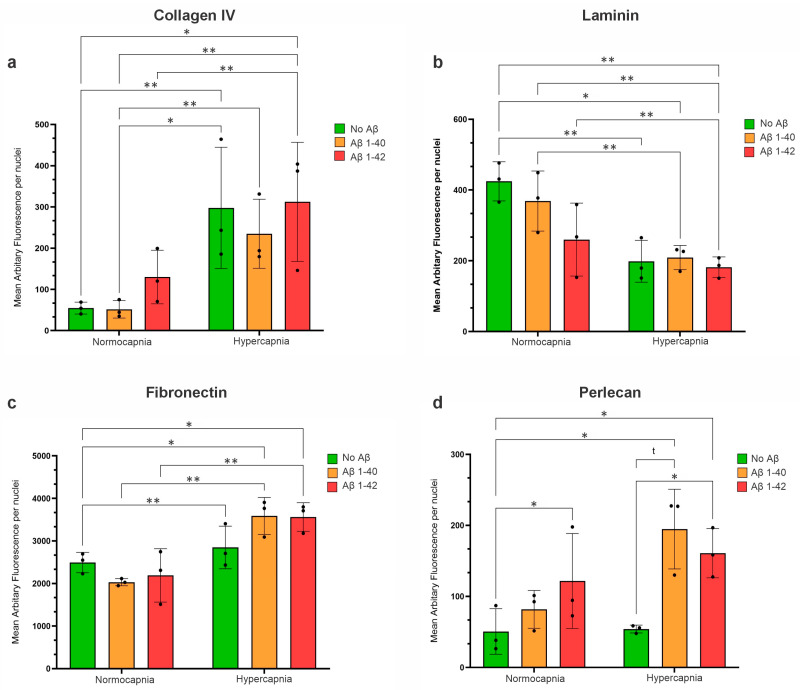
BM protein expression in HBVSMCs cultured under normocapnia or hypercapnia, in the absence or presence of 100 nM Aβ40 or Aβ42. (**a**) Collagen IV expression was significantly increased under hypercapnia conditions. The presence of Aβ40 or Aβ42 did not significantly affect collagen IV levels. (**b**) Laminin expression was significantly decreased under hypercapnia conditions. The presence of Aβ40 or Aβ42 did not significantly affect laminin levels. (**c**) Fibronectin expression was significantly increased under hypercapnia conditions. The presence of Aβ40 or Aβ42 did not significantly affect collagen IV levels. (**d**) Perlecan expression was significantly increased under hypercapnia conditions relative to normocapnia but only in the presence of Aβ40 or Aβ42. HBVSMCs cultured under hypercapnia but in the absence of Aβ showed no change in collagen expression relative to normocapnia. Error bars represent standard deviation. *p <* 0.05 (*), *p <* 0.01 (**). Black dots represent biological replicates (*n* = 3).

To understand how hypoperfusion and ageing can influence the basement membranes of HBVSMCs, cells were cultured for 72 h under normocapnia (5% CO_2_) or hypercapnia (8% CO_2_), in the presence or absence of Aβ 1–40 or Aβ 1–42. Quantification of the immunofluorescence showed that hypercapnia in the absence or presence of Aβ 1–40 or Aβ 1–42 increased the levels of collagen IV deposition (*p* = 0.0031) in HBVSMCs (Figure 4a). The presence of Aβ 1–40 and 1–42 alone did not significantly affect collagen IV levels.

#### 3.7.1. Hypercapnia Decreased Laminin Expression in HBVSMCs

Hypercapnia, in the presence or absence of Aβ 1–40 or Aβ 1–42, decreased laminin expression in HBVSMCs (*p* = 0.004) (Figure 4b). Under normocapnia, the presence of Aβ 1–40 decreased laminin expression relative to cells not exposed to Aβ, and the presence Aβ 1–42 further decreased laminin expression relative to Aβ 1–40; however, these changes were not statistically significant (*p* ≤ 0.05). This pattern was not observed under hypercapnia conditions. Therefore, the presence of Aβ alone did not affect laminin expression in cells cultured under normocapnic or hypercapnic conditions (Figure 4b).

#### 3.7.2. Hypercapnia Increased Fibronectin Expression in HBVSMCs

Hypercapnia, in the absence or presence of Aβ 1–40 or Aβ 1–42, increased the levels of fibronectin in HBVSMCs (*p* = 0.0031) (Figure 4c). Under hypercapnia, the presence of Aβ 1–40 or Aβ 1–42 increased fibronectin levels relative to no Aβ, although this was not statistically significant. Therefore, the presence of Aβ alone did not significantly affect fibronectin expression in cells cultured under normocapnia or hypercapnia (Figure 4c).

#### 3.7.3. Hypercapnic Conditions Increase the Expression of Perlecan in HBVSMCs, but Only in the Presence of Aβ

The presence of Aβ 1–42 increased perlecan expression in HBVSMCs cultured under normocapnia (*p* = 0.0484) or hypercapnia (*p* = 0.0123) conditions relative to cells not exposed to Aβ (Figure 4d). Perlecan was also increased in the presence of Aβ 1–40 relative to no Aβ under hypercapnia, although this was only a trend (*p* = 0.0589). Furthermore, the presence of Aβ 1–40 and Aβ 1–42 under hypercapnia increased perlecan expression relative to the HBVSMC culture under normocapnia in the absence of Aβ (Figure 4d).

## 4. Discussion

In this study, we characterised the composition of BMs secreted in vitro by cerebrovascular cells using human-derived endothelial cells, pericytes, HBVSMCs, and astrocytes. We showed that HBVSMCs secrete significantly higher levels of laminin, perlecan, and fibronectin relative to cerebral endothelial cells, astrocytes, and pericytes, equating to the highest overall BM protein expression, although pericytes expressed significantly greater levels of collagen IV relative to the other cell types. It is unsurprising that HBVSMCs deposit the greatest overall level of BM proteins given the contractility capacity of these cells and their need for adequate anchorage within the complex layers of the arterial wall, although it was surprising that collagen IV, which is the major form of collagen present in the vasculature, was not one of the major BM protein components of these cells [27]. Perlecan was expressed at much lower levels relative to the other BM proteins in each cell type and was found at similar levels in pericytes, astrocytes, and endothelial cells. This is surprising considering the important structural and biochemical function of perlecan in supporting the cerebrovasculature in response to dynamic blood flow [28]. Pericytes and astrocytes also expressed similar amounts of laminin, whilst astrocytes and endothelial cells expressed similar amounts of fibronectin. The unique location of pericytes embedded within the endothelial BM indicate that these cells contribute significantly to the vascular BM [9] and may in fact be important for the induction of BM protein synthesis by ECs [29]. The secretion of collagen IV and fibronectin at relatively high levels in cultured pericytes in our study is consistent with these being major proteins produced by neurovascular pericytes in vivo [30]. The constituents and relative abundance of vascular BMs directly influence their biomechanical properties and therefore the observed differences between relative BM protein compositions across cell types are consistent with their distinct roles within the cerebral vessel wall.

Ageing is associated with the thickening of cerebrovascular BMs, especially in the cortex and hippocampus [5]. Furthermore, thickening of these BMs in the context of CAA and AD has been reported in multiple studies in both human and mouse brains [5,9,31,32,33,34]. BM thickening of cerebral microvessels caused by increased levels of BM proteins in transgenic mice overexpressing TGF-β was observed 5 months prior to the detection of CAA, providing strong evidence that BM thickening precedes and is a significant contributing factor of cerebrovascular Aβ deposition [35]. Stroke and AD share common risk factors, and stroke itself may be a risk factor for AD, suggesting an important relationship between the cerebrovascular changes seen in age-associated pathology and neurodegeneration [9]. In contrast to AD and CAA, animal models suggest that stroke is associated with the degradation of cerebrovascular BMs, caused by the upregulation of proteases following an ischemic event [36,37,38]. We exposed HBVSMCs to elevated CO_2_ to model the hypoperfusion and subsequent hypercapnia that is observed in ageing [39,40], and combined this with Aβ exposure to better understand how the HBVSMC BMs respond to these age-associated conditions and influence downstream pathology.

Our results show increased depositions of collagen IV, fibronectin, and perlecan by HBVSMCs under hypercapnia conditions; however, increased perlecan deposition was dependent on the presence of Aβ, suggesting that whilst elevated CO_2_ alone is sufficient to modify collagen IV and fibronectin, it is the combination of hypoperfusion and Aβ exposure that alters perlecan expression in HBVSMCs. Whilst findings from AD mouse models have been mixed, the levels of collagen IV, fibronectin, and perlecan have consistently been shown to be elevated in BMs from human AD postmortem brains [31,40]. The observed increase in fibronectin is significant in the context of small vessel disease, as fibronectin accumulates in hereditary cerebral autosomal recessive arteriopathy with subcortical infarcts and leukoencephalopathy (CARASIL). Whilst the exact mechanism remains unknown, treatment with the angiotensin II type I receptor antagonist Candesartan has been shown to downregulate fibronectin gene expression in a mouse model of CARASIL [41]. Dysregulated TGFβ signalling has also been proposed in CARASIL, with increased fibronectin expression after experimental stroke in cultured astrocytes and in vivo associated with reactive gliosis and an impaired influx of CSF showing reversal after inhibition of TGFβ [42,43]. These findings suggest that similar mechanisms could be linked to elevated fibronectin in aged and AD vessels. Furthermore, fibronectin has been shown to directly bind Aβ, suggesting that age-related and pathological changes to fibronectin levels in cerebrovascular BMs could directly influence Aβ deposition in vessel walls [43] and CAA risk.

Whilst the presence of Aβ alone did not significantly affect the levels of collagen IV, fibronectin, or laminin, we found that under normocapnia, perlecan levels were increased in the presence of Aβ 1–42. This may be because Aβ 1–42 species are more toxic than Aβ 1–40, in part due to having higher amyloidogenicity and lower solubility than Aβ 1–40 [44,45,46]. Aβ exposure alone also had no effect on laminin deposition; however, we found that hypercapnia decreased laminin levels in HBVSMCs, contrasting the increase observed for the other BM proteins. This supports our previous findings that laminin decreases in vascular BMs in the cortex and striatum of mouse brains with age [5] and in brains from mice with the APOE4 variant [47].

Our choice to use hypercapnia to model ageing was based on the strong relationship between age, arteriolosclerosis, and hypoperfusion, as well as reduced nitric oxide bioavailability, thus responsible for impaired vessel compliance [7]. While there is no direct evidence that hypoperfusion results in hypercapnia, ageing leads to an impaired respiratory response to hypercapnia, as well as reduced cerebrovascular reactivity in response to hypercapnia [22,23]. Furthermore, the major technical limitations of using hypoxia in a cell culture model justify the use of hypercapnia in this study to demonstrate that basement membranes are dynamic entities that respond to changes in blood gases. The findings from this study suggest that hypoperfusion, in combination with other factors characteristic of age-associated cerebrovascular dysfunction not tested in this model, may initiate changes to vessel BMs, including thickening and compositional changes, which encourage the deposition of Aβ. This remodelling of the BM may slow the clearance of Aβ 1–40/42 via IPAD pathways and promote CAA via multiple pathophysiological mechanisms, including (1) altered specific binding of ECM proteins to soluble Aβ40/42, (2) increased tortuosity of the perivascular spaces to the diffusion of interstitial fluid solutes and reduced vascular reactivity due to (3) reduced vessel compliance, and (4) impaired contractility of mural cells.

The molecular mechanisms by which hypoperfusion modulates the BM composition remains unclear. Matrix metalloproteinases (MMPs) mediate the proteolytic cleavage of ECM proteins, and studies have shown that age-related changes including hypoxia, blood–brain barrier dysfunction, inflammation, and oxidative stress induce MMP activity, compromising the functional integrity of the ECM [48,49,50]. This could explain the reduction in laminin levels in HBVSMCs following exposure to hypercapnia in this study, and future studies should quantify MMP levels to further interrogate this. This is also consistent with a study that found that serum MMP-2 and MMP-9 levels were increased in patients with acute ischemic stroke, whilst intact serum laminin was significantly decreased [51]. However, this does not align with the other results from this study and indeed other studies that have shown increased levels of BM proteins in ageing and AD [5,9,31,32,33,34]. Tissue Inhibitor of Metalloproteinases (TIMPs) are secreted extracellular proteins that inhibit the activities of MMPs, and the family member TIMP-3 has a broad MMP inhibitory capacity [52]. Our previous study using quantitative proteomics to investigate protein changes in CAA revealed TIMP-3 to be upregulated in leptomeningeal arteries from CAA patients compared to young and elderly controls. Qualitative immunohistochemical analyses also confirmed increased TIMP-3 levels in elderly vessels versus young controls [53], suggesting that TIMP-3 levels correlate with ageing. These findings were supported by a more recent proteomic study that detected TIMP-3 in CAA vessels, alongside an upregulation of ECM-associated proteins in both CAA and age-matched control vessels [54]. Therefore, future studies should focus on TIMP-3 as a potential key causal protein of vascular BM dysfunction in ageing and CAA, leading to ECM protein accumulation, vessel fibrosis, impaired IPAD.

The finding that Aβ alone did not initiate changes to collagen IV, fibronectin, or laminin suggests that age-associated BM changes are causative rather than correlative of Aβ deposition. However, our study also suggests that certain BM proteins such as perlecan may also be particularly reactive to the presence of Aβ, specifically Aβ 1–42, further disturbing and amplifying age-associated pathological changes in the cerebrovasculature that contribute to CAA. In fact, it has been proposed that an early accumulation of heparan sulphate proteoglycans (HSPGs), of which perlecan is a major type, shows a common link with and is a possible key initiating event in AD pathobiology [55]. The increase in perlecan in the presence of Aβ 1–42 is also important in the context of immunisation protocols against Aβ species, as after Aβ immunisation, there is an increase in vascular Aβ, suggesting a process by which solubilized Aβ plaques are entrapped or retained in the IPAD pathway [56,57], which may subsequently result in changes to cerebral SMC BMs that negatively influence long-term cerebral vessel health and the clearance of Aβ via IPAD.

The highly ordered and web-like expression pattern of collagen IV and fibronectin observed in HVSMSCs was lacking in hCMEC/D3 cell cultures. Whilst these cells have been extensively characterised and are regularly used as a model of the human blood–brain barrier, a limitation of this immortalised cell line relative to the other primary cell types used in this study is the possibility that genetic modifications have influenced their capacity to produce and assemble functional BMs that are representative of in vivo cells. Furthermore, whilst the hCMEC/D3 cell line is derived from temporal lobe microvessels, it is not possible to determine if the vessels were capillaries, venules, or small arterioles, which may influence BM composition. The punctate expression pattern of laminin in astrocytes and pericytes was distinct from the fibrous pattern seen in HBVSMCs and ECs. It has been shown that different isoforms of laminin are present in different cells of the cerebral vasculature, suggesting that they may play distinct functions [58,59,60,61]; however, it was not possible for us to assess individual isoforms of laminin expressed in our cultures. It is important to acknowledge that whilst in vitro cell models are useful tools, they may not fully recapitulate vascular cell behaviour occurring in vivo. It is also important to acknowledge that the distinct media required for each cell type may also influence the production and deposition of BM proteins. Furthermore, hypercapnia and normocapnia environments were achieved using separate cell culture incubators, and as such, it is important to acknowledge this as a possible variable that could have influenced cell behaviour. 

In this study, we focused on the response of HBVSMCs to hypercapnia/Aβ due to the critical role of these cells in driving the clearance of Aβ along BMs of cerebrovascular arterioles via intramural periarterial drainage (IPAD). With ageing, the vasomotive force of HBVSMCs is diminished and their basement membrane is altered, impairing Aβ clearance and contributing to cerebral amyloid angiopathy. Whilst the basement membranes of endothelial cells, pericytes, and astrocytes play import roles in vascular health, their response to ageing in the context of IPAD may not be as critical. However, future studies investigating the effects of hypercapnia and Aβ on these cells would offer a more comprehensive understanding of the influence of these factors on cerebrovascular BM dynamics with age.

Furthermore, the dynamic nature of BM protein secretion is such that a quantitative analysis of protein expression through immunostaining may not be capable of fully capturing the changes to density, composition, and thickness of BM proteins in response to ageing factors; therefore, future studies should also incorporate techniques capable of measuring structural and molecular modifications to BMs. Furthermore, the crosstalk between the different cells is critical in regulating the deposition and maintenance of BM proteins within the brain’s vasculature, which is crucial for maintaining the integrity of the blood–brain barrier and the conduit for the drainage of interstitial fluid and soluble Aβ as IPAD. As such, future studies should investigate the effects of hypoperfusion and Aβ 1–40/42 on all the cells of the cerebrovasculature, and do so in conditions that better mimic the physiology and structure of vascular walls, such as co-cultures of endothelial cells and mural cells and in vitro fluidic systems that simulate interstitial fluid flow [5,62].

## 5. Conclusions

The individual cell types that compose cerebral arteries secrete varying proportions of the BM proteins laminin, perlecan, collagen IV, and fibronectin in vitro, suggesting distinct roles for these cells in contributing to the extracellular composition of cerebral vessels. Furthermore, hypercapnia and Aβ40, the presence of which is associated with vascular ageing, modify the composition of HBVSMCBMs, which may contribute to the failure of IPAD in ageing.

## Data Availability

The datasets generated and/or for this study are available from the corresponding author on reasonable request.

## Abbreviations

The following abbreviations are used in this manuscript:

BMs	Basement Membranes
SMCs	Smooth Muscle Cells
Aβ	Amyloid Beta
IPAD	Intramural Periarterial Drainage
AD	Alzheimer’s Disease
CAA	Cerebral Amyloid Angiopathy
BBB	Blood–Brain barrier
HSPGs	Heparin Sulphate Proteogylcans
HA	Human Astrocytes
HBVSMCs	Human brain Vascular Smooth Muscle Cells
HP	Human Pericytes
hCMEC/D3	Human Cerebral Endothelial Cell Line
PBS	Phosphate-Buffered Saline
BSA	Bovine Serum Albumin
AF	Arbitary Fluorescence
CARASIL	Cerebral Autosomal Recessive Arteriopathy with Subcortical Infarcts and Leukoencephalopathy
MMP	Matrix Metalloproteinases
ECM	Extracellular Matrix
TIMPs	Tissue Inhibitor of Metalloproteinases
